# Management of Ankle Fractures: Does the Timing of Surgical Intervention Have an Impact on Patient Outcomes?

**DOI:** 10.7759/cureus.99924

**Published:** 2025-12-23

**Authors:** Muhammad Muneeb Safdar, James Williams

**Affiliations:** 1 Surgery, Musgrove Park Hospital, Taunton, GBR; 2 Trauma and Orthopaedics, Musgrove Park Hospital, Taunton, GBR

**Keywords:** ankle fracture, delayed surgical intervention, diabetes, smoking, wound complications

## Abstract

Introduction

Orthopaedic surgeons frequently manage patients presenting with an ankle fracture. It has been observed that these injuries can be present in both young and elderly patients. Surgical intervention with open reduction and internal fixation is often performed for unstable ankle fractures.

Methods

This study was performed at a district general hospital in the United Kingdom and is a retrospective cohort study. The electronic database was reviewed from January 2021 to December 2024 to identify and review the notes for the patients to be included in the study. After identifying the patients, they were divided into two groups. The patients who had surgical intervention in less than 10 days were allocated to the early surgery group, and the patients who had surgery at day 10 or after day 10 of the injury were allocated to the delayed surgery group.

Results

Overall, 96 patients were included in this study, where 29 (30.21%) had early surgical intervention and 67 (69.79%) had delayed surgical intervention. The results were not statistically significant for patient outcomes between the two groups. A correlation was observed between the age of the patients and the length of hospital stay.

Conclusion

At present, there is no definitive consensus with regards to the optimal timing for surgical intervention of ankle fractures. The results of this study highlight that there was no significant difference for post-operative complications between the two groups, which is consistent with the literature. A study with a larger sample size is required to analyse the long-term patient outcomes.

## Introduction

Orthopaedic surgeons frequently treat ankle fractures, and they account for approximately 9% of all fractures in adults [1]. It has been observed that the annual incidence of ankle fractures is approximately 174 per 100,000 adults [2]. Additionally, ankle fractures led to 332,617 hospital admissions in England between 2004-2005 and 2013-2014 [3]. Although young adults frequently sustain ankle fractures due to sports injuries or from a high mechanism injury, ankle fractures are also commonly observed in the elderly population due to falls or twisting injuries [4]. It is therefore essential to treat ankle fractures promptly and appropriately to avoid any long-term complications.

The AO Foundation/Orthopaedic Trauma Association (AO/OTA), Danis-Weber, and Lauge-Hansen classifications are frequently used to classify ankle fractures according to the severity of the injury; AO/OTA and Danis-Weber both emphasise the lateral malleolus in relation to ankle syndesmosis [1]. Danis-Weber type A fractures do not require surgical intervention, as they are stable injuries that are distal to the ankle syndesmosis [5]. Type B Danis-Weber injuries are at the level of the ankle syndesmosis and can be treated either surgically or conservatively, depending on the disruption of the ankle syndesmosis [5]. In contrast, type C Danis-Weber injuries are unstable and are treated with surgical intervention as the ankle syndesmosis is compromised [5]. Alternatively, the Lauge-Hansen classification focuses on the deforming forces at the time of injury, guiding clinicians to reduce the fracture [1]. 

Although stable ankle fractures can be managed conservatively, unstable ankle fractures often require surgical intervention. The options for surgical intervention commonly include open reduction and internal fixation (ORIF) of the ankle [6]. An alternative to ORIF is the intramedullary fibula nail, which may be used for patients with an unstable distal fibula fracture [6]. The surgical intervention may be delayed for unstable ankle fractures; this could be due to several reasons, including soft tissue injury or swelling [7]. The ankle may also need to be stabilised temporarily with an external fixator prior to having definitive internal fixation [7].

The guidelines produced by the British Orthopaedic Association (BOA), British Orthopaedic Association Standards for Trauma and Orthopaedics (BOAST), for the management of ankle fractures are helpful for the management of ankle fractures [8]. It recommends that the aim of the surgical intervention should be to stabilise the ankle mortise [8]. The BOAST guidelines also recommend that the surgical intervention should be performed either on the day of or the day after the injury for the majority of patients who are under the age of 60 years [8].

Infection is one of the significant post-operative complications in foot and ankle surgery. Additionally, there is a higher risk of infection with open fractures, high BMI and patients who continue to smoke or drink alcohol [7]. It has been suggested that early surgical intervention for ankle fractures, within 48 hours, may lead to fewer wound complications and a shorter length of hospital stay [9,10]. A study by Singh et al. found that performing surgery within 24 hours led to a shorter hospital stay for patients [11]. It was noted by Laggner et al. that early surgical intervention, within 24 hours of admission, was associated with a lower risk of infection in addition to having a shorter hospital stay [12]. In contrast, the study by Tantigate et al. did not observe a significant increase in post-operative wound complications for patients having ankle ORIF after 14 days [13]. Similarly, Naumann et al. found that the timing of surgery for ankle fractures was not associated with complications or the length of hospital stay [14]. The ideal timing for surgical intervention of ankle fractures is controversial. Since both early and delayed surgical intervention is supported in the literature [11-14], additional research is required to ascertain the optimal time for surgical intervention of ankle fractures. 

The primary aim of this study was to assess whether delayed surgical intervention for ankle fractures would have an impact on patient outcomes, particularly for wound complications and length of hospital stay. The secondary aim was to analyse the factors that may predict post-operative complications. Additionally, we hope to add further evidence to the literature with regards to the management of ankle fractures.

## Materials and methods

This study was a retrospective cohort study conducted in the United Kingdom at a district general hospital. Patients with an ankle fracture were identified through electronic patient records, and they were reviewed from January 2021 to December 2024. Patients aged between 18 and 75 years were included in this study if they had an isolated closed ankle fracture. The exclusion criteria consisted of patients having an open ankle fracture, polytrauma patients, a pilon fracture, patients under the age of 18 years or over the age of 75 years, and patients having delayed ORIF due to the application of an external fixator previously. Patients were also excluded from the study if their ankle fracture was managed conservatively.

The data were recorded on an NHS computer, where Microsoft Excel (Microsoft® Corp., Redmond, WA, USA) was used. The electronic database was reviewed to record the data regarding the patient’s age, date of injury, type of ankle fracture, the date of them first being reviewed in an outpatient clinic, date of surgery, whether they had diabetes, any post-operative wound complications, any delayed union or non union, their past medical history, any other post-operative complications, length of hospital stay and whether they smoke. The clerking notes at the time of admission and outpatient clinic letters were reviewed to gather this information for the patients. Additionally, the date of the injury was confirmed through the X-rays that demonstrated the ankle fracture. As this was a retrospective cohort study, the X-rays were already reported at the time they were reviewed for this study. For post-operative complications that excluded wound complications, this study reviewed whether there were any secondary operations due to failure of the fixation or metalwork that was symptomatic and required subsequent removal. 

The patients were subsequently divided into two groups following the data collection. The first group consisted of patients who had surgical intervention for their ankle fracture in less than 10 days of the injury (early surgery group). The second group consisted of patients who had surgical intervention at day 10 or after 10 days of the injury (delayed surgery group). The patient outcomes were compared for these two groups. 

For this study, Statistical Package for the Social Sciences (SPSS) version 30 (IBM Corp., Armonk, NY, USA) was used to analyse the data. The Shapiro-Wilk test was used to assess the normality of the data. Additionally, Fisher's exact test was used to analyse the categorical data, and the Mann-Whitney U test was used to analyse the nonparametric data. We also used a regression test to observe any correlations for complications and the length of inpatient stay.

## Results

After reviewing the electronic database, a total of 96 patients were included in this study. Out of the 96 patients, 29 (30.21%) had surgical intervention in less than 10 days (early surgery group), whereas 67 (69.79%) had surgical intervention at day 10 or after day 10 of the injury (delayed surgery group). The demographic data is shown in Table 1. In our cohort, 5 patients (5.21%) had diabetes, and 29 patients (30.21%) had a history of smoking. The patients presented with different types of ankle fractures, as described in Table 1. It was observed that a significant proportion of the patients, 57 patients (59.38%), had a trimalleolar ankle fracture (59.38%).

**Table 1:** Demographic data and characteristics of the patients * Mann-Whitney U test; ^ Fisher's exact test

	Early surgery (n=29)	Delayed Surgery (n=67)	P value
Mean age (SD)	50.76 (15.24)	47.00 (17.01)	0.344*
Diabetes, n (%)	1 (3.45%)	4 (5.97%)	1^
History of smoking, n (%)	5 (17.24%)	24 (35.82%)	0.091^
Type of ankle fracture, n (%)	29 (30.21%)	67 (69.79%)	0.122^
Danis-Weber type B, n (%)	4 (13.79%)	7 (10.45%)	
Danis-Weber type C, n (%)	4 (13.79%)	2 (2.99%)	
Bimalleolar, n (%)	5 (17.24%)	16(23.88%)	
Trimalleolar, n (%)	15 (51.72%)	42 (62.69%)	
Isolated posterior malleolus, n (%)	1 (3.45%)	0 (0%)	

The patient outcomes for the two groups are described in Table 2. Although the wound complications were higher in the delayed surgery group, where seven patients (10.45%) had wound complications, the results were not statistically significant. Additionally, only 2 patients (6.70%) had wound complications in the early surgery group. Whilst the data was being analysed, it was observed that none of the patients had delayed union or non-union for the two groups. Despite the median length of hospital stay being one day for both groups, the results were not statistically significant, where the p value was 0.053, as described in Table 2.

**Table 2:** Patient outcomes according to the timing of surgical intervention ^ Fisher's exact test; * Mann-Whitney U test - P value was not calculated due to no patients having delayed union or non-union

	Early surgery (n=29)	Delayed Surgery (n=67)	P value
Wound complications, n (%)	2 (6.70%)	7 (10.45%)	0.719^
Post-operative complications excluding wound complications, n (%)	5 (17.24%)	7 (10.45%)	0.502^
Delayed union, n (%)	0 (0%)	0 (0%)	-
Non-union, n (%)	0 (0%)	0 (0%)	-
Median length of hospital stay (SD)	1 (3.053)	1 (3.526)	0.053*

In addition to the wound complications, we also analysed whether the patients had any other post-operative complications, excluding wound complications. The following other post-operative complications were observed in this study: removal of metalwork, complex regional pain syndrome, ankle arthroscopy due to ongoing symptoms and arthrodesis due to post-traumatic arthritis. These post-operative complications were noted to be higher in the delayed surgery group compared to the early surgery group. There were 5 patients (17.24%) in the early surgery group who had these complications, whereas the delayed surgery group had 7 patients (10.45%) who experienced these complications. The results for these two groups were not observed to be statistically significant, as described in Table 2.

To assess for any correlation between the outcomes, a multiple regression test was performed, which is described in Table 3. The dependent variables were wound complications and post-operative complications, excluding wound complications and length of hospital stay. It was observed that there was no significant relationship between wound complications and the independent variables, as mentioned in Table 3. There was a correlation between post-operative complications, excluding wound complications and smoking, with a p value of 0.0158. A correlation was also observed between age and length of hospital stay, where the p value was 0.004. Furthermore, a correlation was observed between diabetes and the length of hospital stay.

**Table 3:** Multiple linear regression test * Statistically significant p value

	Wound complications p value	Post-operative complications, excluding wound complications p value	Length of hospital stay p value
Age	0.145	0.428	0.004*
Smoking history	0.194	0.0158*	0.716
Diabetes	0.408	0.391	0.037*

## Discussion

The results of this study are in keeping with the literature. Most of the patients in this study were in the delayed surgery group, where 67 patients (69.79%) had delayed surgical intervention. Although diabetes is a well-known risk factor for post-operative complications, particularly in lower limb surgery, only five patients (5.21%) had diabetes in this study. 

Several factors can contribute to the surgical intervention being delayed for patients presenting with an ankle fracture. If there is a fracture dislocation, a delayed reduction of the ankle joint can lead to significant soft tissue swelling that could cause a delay in subsequent surgical intervention. The patients may also require medical optimisation perioperatively prior to the surgical intervention. Furthermore, additional imaging, such as a CT scan, may not be performed out of hours, and this could lead to a delay in pre-operative planning and surgical intervention. The availability of a foot and ankle surgeon can also affect the timing of surgical intervention, as complex ankle fractures often require specialist input.

In our study, 21 patients (21.88%) experienced post-operative complications, where 9 patients (9.38%) had wound complications post-operatively, and 12 patients (12.5%) had post-operative complications excluding wound complications. Macera et al. conducted a large study of 378 patients, and they observed that 36% of their patients experienced post-operative complications [15]. This is in keeping with our study, where 21 patients (21.88%) had post-operative complications. Although the rate of post-operative complications following surgical intervention for ankle fractures varies in the literature, complication rates of up to 40% have been observed [6-18]. The results of this study are comparable with the post-operative complications rates in the literature.

In addition to the post-operative complications being in keeping with the literature, the results of this study did not observe a significant difference in wound complications between the early and delayed surgical groups. Similarly, there was no significant difference in post-operative complications, excluding wound complications, between the two groups. The study by Kim et al. also found no significant difference related to the timing of surgical intervention and wound complications [19]. This was a large study of 296 patients where the patients were divided into three groups according to the timing of surgical intervention [19]. Similarly, Naumann et al. did not observe a significant difference in post-operative complications in relation to the timing of surgery [14]. However, a significant difference between the timing of surgical intervention and post-operative complications has also been observed in the literature. The study by Schepers et al. noted the wound complications to be significantly greater for patients having surgery after 24 hours and one week compared to those having surgery within 24 hours and one week [10]. It has also been observed that delayed surgery for Danis-Weber type B fractures can be associated with frequent post-operative infections [10]. Nevertheless, the wound and other post-operative complications have been comparable for the early and delayed surgery groups in this study.

In this study, we observed that the median length of hospital stay was the same for the early and delayed surgery groups, and the results were not statistically significant. In contrast, the study by Singh et al. found that the mean length of post-operative hospital stay was significantly shorter for the early surgery group compared to the late surgery group [11]. In this study, we did not analyse the impact of post-operative rehabilitation and physiotherapy on the length of hospital stay. It is possible that a delay in rehabilitation could lead to a longer hospital stay for the patients, which could potentially contribute to the results of this study not being statistically significant.

Through the multiple regression tests, a correlation was observed between the age of patients and the length of hospital stay. Elderly patients are likely to have a higher American Society of Anesthesiologists (ASA) grade due to their comorbidities, which could lead to a longer length of hospital stay due to a prolonged post-operative recovery period. In a study by Yang et al., an association was observed between age, comorbidities, and length of hospital stay, along with the cost of stay [20]. Additionally, we observed an association between smoking and post-operative complications, excluding wound complications. This is in keeping with the literature where Jafar et al. reported that smoking significantly increases the risk of complications in patients having foot and ankle surgeries, which includes wound infections and non-union [21].

This study only had 29 patients (30.21%) in the early surgery group. If a different time frame had been selected for this study, the patients would likely have been evenly distributed between the two groups. This could have subsequently had a potential impact on patient outcomes being statistically significant.

It is also essential to highlight that the delayed surgical intervention for ankle fractures is often due to theatre capacity. Whilst it would be ideal for the patients to have surgical intervention for ankle fractures within the recommended time frame by BOAST guidelines [8], other injuries often take priority; these injuries include hip fractures, open fractures and paediatric fractures. Due to limited theatre capacity, surgical intervention for ankle fractures is often delayed because of necessity rather than choice.

The major limitation of this study was the small sample size. As the patients are not evenly distributed between the two groups due to the time frame selected, a larger sample size would help to support the results. The authors of this study reviewed the results retrospectively, and it would have been ideal to conduct a prospective study. By only including patients under the age of 75 for this study, the authors attempted to minimise the risk of post-operative complications caused by other co-morbidities. Additionally, the impact of delayed surgical intervention on long-term outcomes was not assessed in this study. The impact of delayed rehabilitation on the length of hospital stay was also not assessed in this study; this could be assessed in future studies.

## Conclusions

Overall, the results of this study are consistent with the literature. This study highlights the significance of timing for surgical intervention for patients presenting with an ankle fracture. The results of this study emphasise that the length of hospital stay could be affected by the age of the patient, and this is something that could be addressed during the peri-operative period by optimising the co-morbidities. Furthermore, this study highlighted the impact of smoking on post-operative complications; additional support related to smoking cessation can reduce the risk of post-operative complications. Although there is no definite consensus on the timing of surgical intervention for ankle fractures, the results of this study can help clinicians in the decision-making process. A study with a larger sample, analysing the long-term outcomes, is required to determine the definitive time for surgical intervention of ankle fractures.
